# Environmental factors influencing older adults’ walking for transportation: a study using walk-along interviews

**DOI:** 10.1186/1479-5868-9-85

**Published:** 2012-07-10

**Authors:** Jelle Van Cauwenberg, Veerle Van Holle, Dorien Simons, Riet Deridder, Peter Clarys, Liesbet Goubert, Jack Nasar, Jo Salmon, Ilse De Bourdeaudhuij, Benedicte Deforche

**Affiliations:** 1 Department of Human Biometry and Biomechanics, Faculty of Physical Education and Physical Therapy, Vrije Universiteit Brussel, Pleinlaan 2, B-1050, Brussels, Belgium; 2 Department of Movement and Sport Sciences, Faculty of Medicine and Health Sciences, Ghent University, Watersportlaan 2, B-9000, Ghent, Belgium; 3 Department of Experimental – Clinical and Health Psychology, Faculty of Psychology and Educational Sciences, Ghent University, Henry Dunantlaan 2, B-9000, Ghent, Belgium; 4 City and Regional Planning, The Ohio State University, 230 Knowlton Hall, Columbus, OH, 43210, USA; 5 School of Exercise & Nutrition Sciences, Deakin University, Burwood Highway 221, Burwood, VIC, 3125, Australia

**Keywords:** Physical environment, Physical activity, Walking for transportation, Older adults, Qualitative study, Walk-along interviews

## Abstract

**Background:**

Current knowledge on the relationship between the physical environment and walking for transportation among older adults (≥ 65 years) is limited. Qualitative research can provide valuable information and inform further research. However, qualitative studies are scarce and fail to include neighborhood outings necessary to study participants’ experiences and perceptions while interacting with and interpreting the local social and physical environment. The current study sought to uncover the perceived environmental influences on Flemish older adults’ walking for transportation. To get detailed and context-sensitive environmental information, it used walk-along interviews.

**Methods:**

Purposeful convenience sampling was used to recruit 57 older adults residing in urban or semi-urban areas. Walk-along interviews to and from a destination (e.g. a shop) located within a 15 minutes’ walk from the participants’ home were conducted. Content analysis was performed using NVivo 9 software (QSR International). An inductive approach was used to derive categories and subcategories from the data.

**Results:**

Data were categorized in the following categories and subcategories: access to facilities (shops & services, public transit, connectivity), walking facilities (sidewalk quality, crossings, legibility, benches), traffic safety (busy traffic, behavior of other road users), familiarity, safety from crime (physical factors, other persons), social contacts, aesthetics (buildings, natural elements, noise & smell, openness, decay) and weather.

**Conclusions:**

The findings indicate that to promote walking for transportation a neighborhood should provide good access to shops and services, well-maintained walking facilities, aesthetically appealing places, streets with little traffic and places for social interaction. In addition, the neighborhood environment should evoke feelings of familiarity and safety from crime. Future quantitative studies should investigate if (changes in) these environmental factors relate to (changes in) older adults’ walking for transportation.

## Background

Despite the well-known physical, mental and social health benefits of regular physical activity (PA), most older adults are insufficiently active [1-4]. Walking is an ideal activity for this population as it is safe, accessible and well-liked [5]. Especially, walking for transportation seems promising as people can easily make it part of their daily routine (e.g. walking to a shop). Promoting walking for transportation requires the knowledge of its correlates [6]. Despite the strong focus on individual correlates earlier, socio-ecological models emphasize the importance of the individual’s interactions with the surrounding physical, social and policy environment [7-9].

The physical environment is defined as objective and perceived characteristics of the physical context in which people spend their time (e.g. home, neighborhood), including aspects of urban design (e.g. presence of sidewalks), traffic density and speed, distance to and design of venues for PA (e.g. parks), crime, safety and weather conditions [10]. Physical barriers (e.g. distance, slopes, obstacles…) might especially hinder PA in older adults as age-related functional limitations and fear of falling can cause difficulties in overcoming these barriers [11-13]. That said, while a systematic review retrieved 32 quantitative studies on the relationship between the physical environment and older adults’ PA, these studies yielded inconsistent results, the majority of studies were conducted in the US and only six focused on walking for transportation [14]. Furthermore, the mechanisms underlying the interaction between the objective and perceived environment needs further exploration [15-17].

Few studies used qualitative methods, even though such methods offer a way to find out not only what but also how and why environmental factors relate to PA [18]. As qualitative methods aim to understand the meaning of an experience to participants in a natural setting using systematic observations and interactional methodologies [19], it can provide insight into the interaction between the objective and perceived environment for walking. Furthermore, it can identify new environmental factors and more detailed information on previously studied factors. Resea`rchers could use such information to refine tools for measuring the environment, tools which might previously have failed to find consistent and statistically significant relationships to older adults’ PA [14].

The few qualitative studies available have used in-depth one-on-one interviews [20], group interviews [21,22], a combination of those [23], or a “photovoice” methodology. In this latter older adults took photographs of environmental supports and barriers and discussed them in facilitated focus group interviews [24,25]. Because all of these qualitative studies took place in Northern America, it is uncertain if their findings apply elsewhere. Furthermore, although previous qualitative studies provided rich information, their reliance on indoor sit-down interviews discourages context-sensitive reactions of the interviewer and interviewee. These methods also require a level of cognitive awareness of the situations/perceptions at the time of exposure to the neighborhood which may not be easily recalled by participants. The photovoice approach may reduce this problem, but it may miss the richness of neighborhood walks in which participants discuss their experiences and perceptions while interacting with and interpreting the local social and physical environment “in the moment” [26,27], in short a “walk-along interview”. In a walk-along interview, the researcher accompanies a participant on a walk in an environment familiar to them, such as their neighborhood. Used by urban planners and sociologists [26] walk-along interviews have particularly value for studying perceptions of and spatial practices in the physical and social environment [27]. Responses from individuals in and moving through real environments have greater ecological validity than would traditional interviews or surveys. Consider one example, Moles [28] used walk-along interviews to study the cultural and social meanings of a park. The researcher approached park visitors and walked through the park with them. During the walks, participants passed by and talked about spots where they gathered and chatted with friends or discussed historical events when encountering monuments. The three way interaction between place, researcher and participant revealed new themes that would not have emerged from traditional interviews. From the above, walk-along interviews seem a promising strategy to investigate environment-PA relationships, however they have not been previously applied with this objective.

To address the scarcity of environment-PA research in older adults and especially qualitative and European studies, the current study aimed to uncover the environmental factors influencing Flemish older adults’ walking for transportation. Because it sought to get detailed and context-sensitive information, it used a qualitative approach with walk-along interviews.

## Methods

### Participants

Purposeful convenience sampling was used to recruit 57 older adults stratified by gender. To be included, participant had to be over 65 years old, dwelling in the community and able to walk independently for at least 30 minutes. Participants were recruited in urban (>600 inh./km²) and semi-urban municipalities (300–600 inh./km²) [29] nearby the cities of Ghent (1558 inh./km²), Antwerp (2364 inh./km²) and Halle (811 inh./km²). Participants were recruited from these three different regions in order to provide sufficient environmental variation.

### Protocol and measures

The protocol had two parts: a structured interview during a home visit and a semi-structured walk-along interview during a walk to a destination in the participant’s neighborhood. During the home visit, a short interview assessed demographics, functional limitations, PA and distance to facilities. Functional limitations were measured using the physical functioning scale of the validated Short-Form 36-item Health Survey [30,31]. An adapted section (a separate question targeting cycling for recreation was added) of the IPAQ (long form, last 7 days, interview version) was used to assess PA. The IPAQ has been validated in older adults [32] and has been used in several previous studies in older adults [33-35]. To examine distance to facilities, the “Stores, facilities and other things in your neighborhood” section of the Neighborhood Environment Walkability Scale (NEWS) [36] was used and extended with destinations relevant for older adults (e.g. friend’s home, senior center). Based upon this last question a destination within a 10 – 15 minutes’ walk was randomly chosen for the walk-along interview.

Before beginning the walk, the researcher read the following instructions: ‘We will now walk to “destination X”. The purpose is that you tell us which things in the environment facilitate or hinder your walking for transportation. Consider things in the environment that facilitate or hinder walking or things that make the walk more or less comfortable, pleasant or interesting. Also consider things that influence your feelings of safety. This can include safety from traffic and safety from crime, but also safety of being injured. Thus, think about all positive and negative things in the environment that influence how you experience the walk. You are the expert and it is the purpose that you tell us freely about your experiences, ideas and opinions, so that we can learn about the things in the environment that facilitate or hinder your walking. Therefore, we might ask some additional questions to completely understand your experiences, ideas and opinions. All the information gathered will be strictly confidential and will only be used for our research. All things that you talk about, will be photographed. Is everything clear for you? As it is too difficult to write down the complete interview, it will be tape recorded. Do you agree with this?’

Additionally, participants were told to disregard walking for recreation and only consider walking for transportation. The participant and researcher walked to and from the destination along two different routes. The route to the destination was the route that the participant would usually follow when walking to this destination. The route back from the destination was chosen by the researcher based upon the availability of different routes resulting in a walk of approximately 30 minutes using a map of the participant’s neighborhood. The study used two different routes to increase the number of different environmental stimuli encountered during the walk-along interview. Additionally, this provided the participants with environments they did not habitually walk along.

While walking, participants were prompted with instructions similar to those given before the walk. Follow-up questions were asked to gather more details about how the factors facilitate or hinder walking. In the case of barriers, suggestions for improvement were inquired. As described in the standardized instructions, photographs were taken to illustrate the environmental factors discussed during the walk. Data collection was performed by four trained researchers (DS, RD, VVH and JVC) during daytime in the period November 2010 - February 2011. The study protocol was approved by the ethical committee of the university hospital.

### Data analyses

Data obtained by the structured interview were entered into a SPSS-file (version 19.0) to calculate descriptive statistics. Data from audiotapes were transcribed verbatim and corresponding photographs were added. NVivo 9 software (QSR International) was used to analyze the data. Data analysis was guided by grounded theory which is characterized by intensively analyzing data, often sentence by sentence, or phrase by phrase [37]. Through constant comparisons, the analysis derived categories and subcategories from the data. When this uncovered categories similar to those in previous studies, they were named according to the categories of the Neighborhood Environment Walkability Scale (NEWS) to allow comparison between studies. The NEWS is the most frequently used questionnaire to assess environmental perceptions [7,36,38]. Analyses were carried out by DS, RD and JVC. Doubts or disagreements were discussed with a fourth researcher (BD) until consensus was reached.

As suggested by Sandelowski (2001) [39] the qualitative data are reinforced by quantitative counts of the participants discussing a certain environmental factor. Thus, when a factor was discussed by less than 25%, we called it “few”, for between 25% and 50%, we called it “some”, for between 50% and 75%, we called it “a lot of” and for more than 75% of the participants, we called it “almost all” in the results’ description (see Table 1).

**Table 1:** Percentages of participants discussing a subcategory and corresponding pronouns used in results’ description

**% of participants discussing a (sub)category**	**Pronoun**
% < 25	Few
25 ≤ % < 50	Some
50 ≤ % < 75	A lot of
% ≥ 75	Almost all

## Results

### Descriptives

Demographics and PA behaviors of the 30 male and 27 female participants are presented in Table 2.

**Table 2:** Descriptive statistics

**Demographics**	
Age (M ± SD)	73.4 ± 5.4
Years at current address (M ± SD)	28.0 ± 15.5
% female	47.4
% higher education	47.4
% car ownership	78.9
% living with a partner	71.9
**PA behaviors** (min/week)	
Walking for transportation (M ± SD)	98.3 ± 122.0
Cycling for transportation (M ± SD)	26.9 ± 52.3
Walking for recreation (M ± SD)	71.6 ± 94.5
Cycling for recreation (M ± SD)	28.4 ± 137.4
Other recreational PA (M ± SD)	40.8 ± 79.5

M = mean, SD = standard deviation, PA = physical activity.

### Content analysis

**Figure 1:**
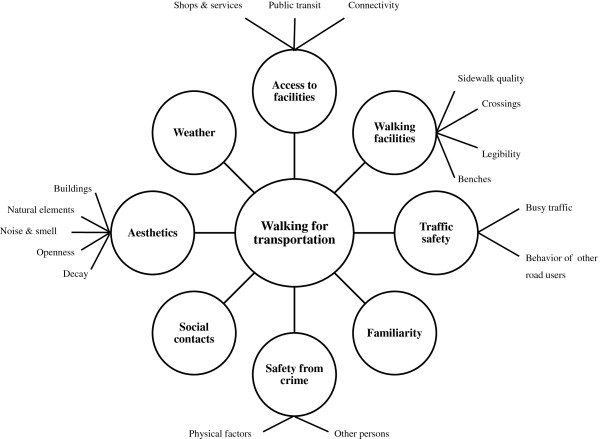
Overview of the categories and subcategories of environmental factors affecting walking for transportation

Qualitative data analysis revealed eight categories of environmental factors that affected walking for transportation: access to facilities (including shops and services, public transit and connectivity), walking facilities (including sidewalk quality, crossings, legibility and benches), traffic safety (including busy traffic and other road users), familiarity, safety from crime, (including physical factors and other persons), social contacts, aesthetics (including buildings, natural elements, noise and smell, openness and decay) and weather (see Figure 1). Table 3 presents the percentages of participants that discussed a certain environmental category.

**Table 3:** Percentages of participants that discussed an environmental category

**Environmental category**	**Total sample**	**Male**	**Female**
	**N = 57**	**N = 30**	**N = 27**
**Access to facilities**			
Shops & services	63.2	53.3	74.1
Public transit	14.0	6.7	22.2
Connectivity	10.5	6.7	14.8
**Walking facilities**			
Sidewalk quality	86.0	80.0	92.6
Crossings	40.4	40.0	40.7
Legibility	7.0	10.0	3.7
Benches	3.5	3.3	3.7
**Traffic safety**			
Busy traffic	86.0	86.7	85.1
Behavior other road users	43.9	53.3	33.3
**Familiarity**	21.0	10.0	33.3
**Safety from crime**			
Other Persons	40.3	26.7	55.6
Physical factors	33.3	20.0	48.1
**Social contacts**	59.6	53.3	66.7
**Aesthetics**			
Buildings	36.8	26.7	48.1
Natural elements	66.7	73.3	59.3
Noise & smell	33.3	36.7	29.6
Openness	24.6	30.0	18.5
Decay	42.1	50.0	33.3
**Weather**	26.3	26.7	25.9

#### Access to facilities

##### Shops & services

A lot of participants stated that the nearby presence of non-residential uses, such as grocery stores, supermarkets, butchers, bakeries, banks, post offices, bars and pharmacies encouraged them to walk to these destinations.

Participants living in city centers reported that they liked living in the city center because all facilities are within a short walk. However, participants also complained about the absence or disappearance of local businesses making it impossible for them to walk to these shops or services.

##### Public transit

Few participants specifically mentioned access to public transit as being a facilitator of walking for transportation. Good access to public transit enabled participants to bridge larger distances than they could by walking alone.

##### Connectivity

Few participants mentioned that they liked to be able to vary their walking route to a destination. Others stated that the presence of pedestrian pathways facilitated walking for transportation by shortening distances to destinations.

### Walking facilities

#### Sidewalk quality

Almost all participants mentioned the importance of the presence and quality of sidewalks. In case of absence of a sidewalk, characteristics of the streets and their shoulders were discussed. Streets with busy traffic or an uneven surface were perceived as less attractive to walk on. When a shoulder was present to walk on, uneven or muddy surfaces were disliked as well. For example, a 69-year-old woman, capturing concerns for busy traffic and shoulders, said:

"“Here you have to walk on the street, that’s not ideal. The only advantage you have, is that traffic is not busy in this street. Otherwise, you couldn’t walk at all. Because when you walk in that street without sidewalks over there, you’re forced to walk on the shoulder because the traffic is very busy. Walking in that grassy shoulder is not easy.”"

When sidewalks were present, almost all participants mentioned issues related to the sidewalks’ quality. They said they liked sidewalks that were well-maintained and even, and judged as hazardous and thus disliked cracked or uneven sidewalks, or sidewalks that had puddles, ice, snow, mud, or leaves. They also viewed sidewalks with steep cross-slopes as hazardous of becoming slippery during snowy and icy conditions. Adequate street lighting was mentioned as important for identifying fall hazards during walks after dark.

Sidewalk width was also discussed. Participants preferred sidewalks wide enough for people to walk next to each other, to easily pass with a wheelchair and to maintain a safe distance from cars. To them, width means usable or walkable width. Walkable width narrows when a sidewalk has construction, parked cars, unkempt greenery and utility or light poles on it, all of which evoked negative responses.

**Figure 2:**
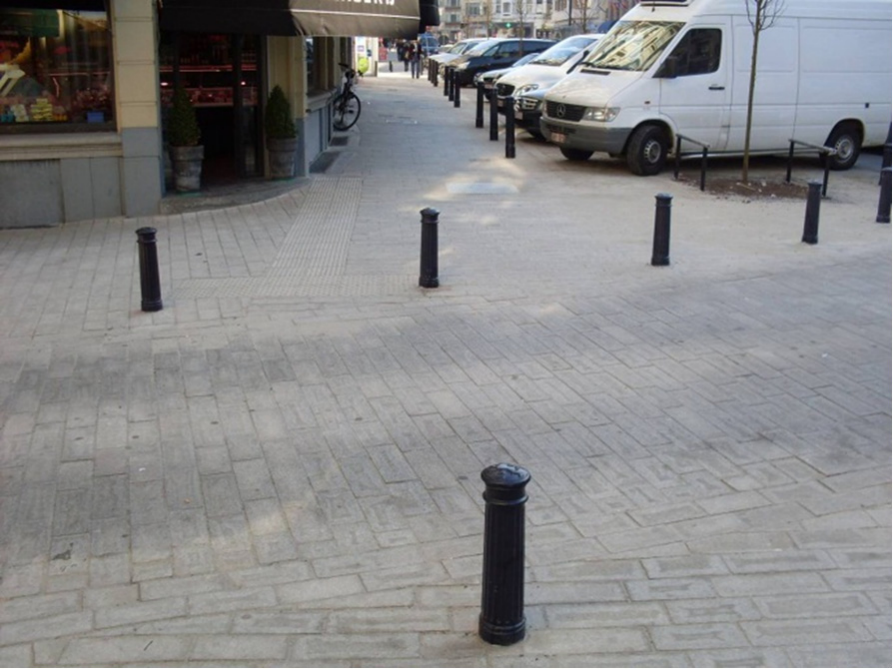
An even sidewalk with bollards separating it from traffic.

Separation of the sidewalk from motorized traffic by parked cars, bollards or vegetation was perceived as positive. Lastly, they said they disliked sidewalks that had high ramps to get on or off, slopes or stairs, because these elements increased the difficulty of walking. A 65-year-old woman reflected concerns for well-maintained sidewalks, absence of ramps and separation from traffic:

"*“At least here you can walk without falling or spraining your ankle, this is all flat. Furthermore, the sidewalk is separated by bollards. If these bollards weren’t here, cars would be parked on the sidewalk* (see Figure 2)*.”*"

#### Crossings

The presence of safe crossings was mentioned by some participants. Zebra crossings, supplemented with traffic lights in busy streets, were considered necessary to be able to cross streets safely. Participants reported to deviate from their shortest route in order to use a zebra crossing or traffic light to safely cross the street.

#### Legibility

**Figure 3:**
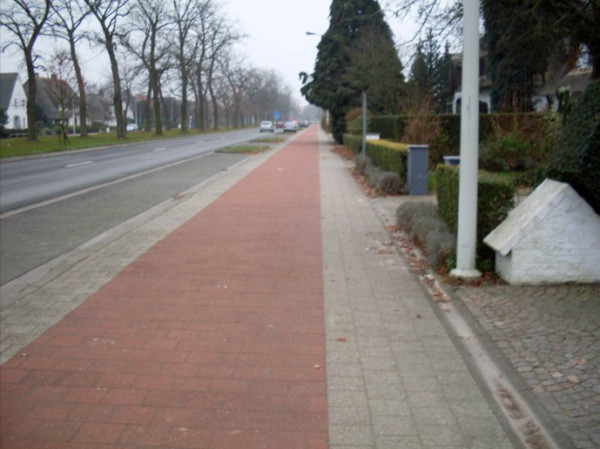
Red tiles clearly marking the cycling path.

Few participants mentioned that poor legibility makes streets less safe and consequently less attractive to walk. Issues discussed in this category concerned confusing traffic rules or indistinct separation between sidewalks and cycling paths. For example, a 76-year-old man, evaluating a recent separation of sidewalk and cycling path, stated:

"*“Recently, they have renewed the sidewalks over here. The situation was really bad. Now it’s better with those red tiles marking the cycling path. Cyclists know where to cycle now. Before, everything was mixed up* (see Figure 3)*.”*"

#### Benches

Few participants mentioned that the presence of benches (would) make the environment more attractive to walk as they could sit down to rest on them.

#### Traffic safety

##### Busy traffic

Almost all participants reported avoiding busy streets and junctions and preferred walking along streets with little traffic, such as residential neighborhoods and cul-de-sacs, because they are safer and less noisy. In this respect, participants also complained about car drivers using shortcuts causing traffic to be busy in small and normally quiet streets.

##### Behavior of other road users

Some participants expressed safety concerns related to the behaviors of other road users. Participants liked streets with slow traffic and disliked streets with speeding cars. This topic was mostly discussed near street crossings, especially when approaching cars were not visible (e.g. near sharp turns). Participants proposed solutions like speed bumps and chicanes to slow down traffic. On the other hand, participants also mentioned car drivers being very courteous and giving priority to pedestrians at crossings.

**Figure 4:**
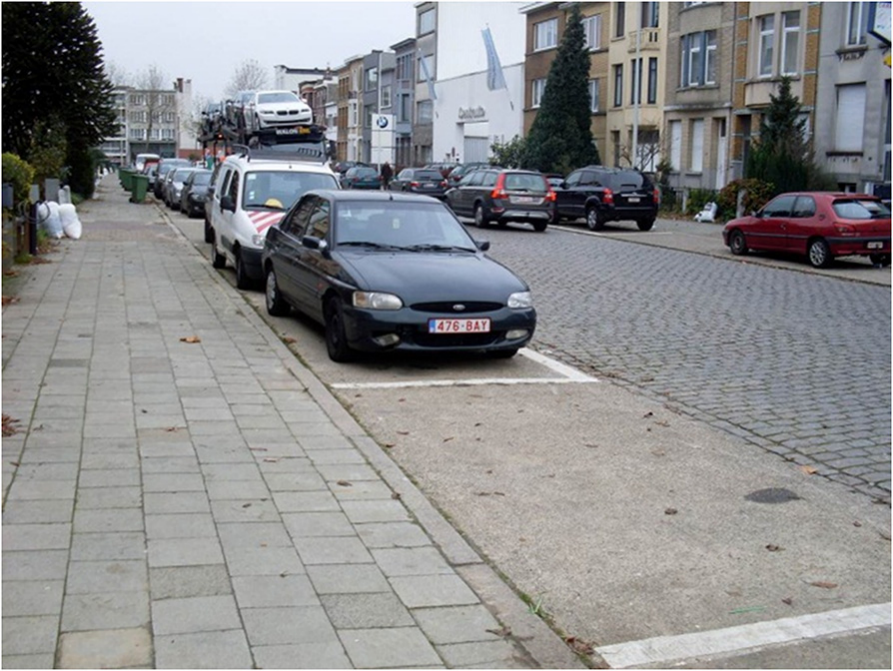
A sidewalk often used by careless cyclists.

Not only speeding cars were disliked but careless cyclists on sidewalks were mentioned as dangerous as well. This is illustrated by the statement of a 66-year-old man:

"*“… the adult cyclists, they just ride on the sidewalk. Possibly, because of the bad condition of the street. But they should at least be careful. Most cyclists ride like they’re on a highway. Older persons are frightened or have to step aside* (see Figure 4)*.”*"

#### Familiarity

Few participants mentioned that they like to walk in streets they are familiar with. Familiar streets provided them with senses of safety and nostalgia. For example, a 78-year-old man mentioned:

"*“It is pleasant to walk here because this was “our” neighborhood in the past, which we know better because we have lived here. You can see the changes, the new buildings and houses, the evolution of the neighborhood…”*"

#### Safety from crime

##### Other persons

Some participants discussed the presence of other persons influencing their perceived safety from crime and therefore their walking experience. After dark participants disliked and avoided walking in abandoned streets, they liked the presence of other persons. A 75-year-old man talked about walking around in the city center during the evening:

"*“The only problem is that around six or seven p.m., the city center is dead. So we won’t go out anymore. During summer there are a lot of people on the terraces. But during this weather, it is dead at six or seven pm. Traffic is not allowed anymore, so people don’t come. I’m always in a hurry to get home because there’s so little movement out here.”*"

However, participants also mentioned that the presence of youngsters, immigrants, beggars and homeless persons made them feel unsafe.

##### Physical factors

Generally, participants felt safe walking in their neighborhood during the day. However, in the evening some participants mentioned feeling unsafe in unlit areas. Especially, abandoned alleys or streets with hiding places for potential offenders (e.g. corners, bushes…) were perceived as unsafe.

#### Social contacts

Participants did not like being alone on the street, they preferred others to be present as well. Participants often met friends or neighbors during their walks and said to enjoy these social contacts. Just saying “hello” or having a short chat made their walks more pleasant. On the other hand, places that were too crowded were disliked. The presence of young children and youngsters was also enjoyed, because it reminded them of their own youth. However, the presence of younger people displaying antisocial behavior (e.g. ignoring traffic rules) was disliked.

#### Aesthetics

##### Buildings

**Figure 5:**
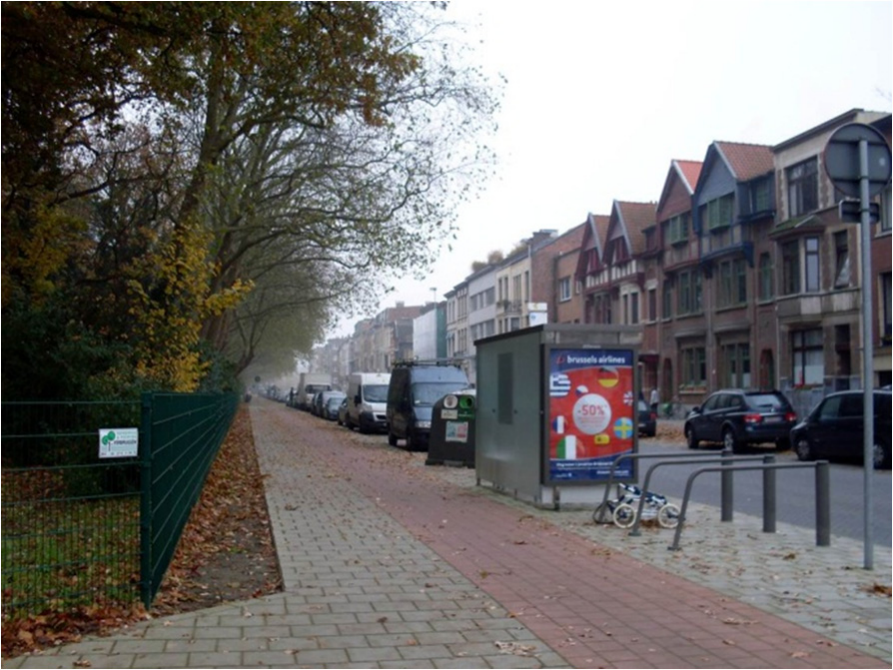
A street with a park on the one side and old, beautiful houses on the other side.

Some participants stated that the presence of certain types of buildings influenced their walking for transportation. They liked walking along historic buildings and monuments. Old houses in a particular style (e.g. art deco) also made streets attractive for walking. For example, an 80-year-old man mentioned:

"*“Here it’s getting more interesting to walk, you have the park on the one side and some very beautiful houses on the other side. These are all from the beginning of the last century and I really like some of them* (see Figure 5)*.”*"

On the other hand, participants stated that they preferred new and well-maintained houses above older houses. The presence of shops was also mentioned as making a walk more pleasant, not only for convenience, but also by making streets more attractive especially when they have nice show windows.

##### Natural elements

A lot of participants said that they really enjoyed the presence of greenery along the route. They liked naturalistic environments because they perceive them as quiet, peaceful and healthy. Parks, fields, woods, rivers/canals/ponds and field tracks were places participants enjoyed passing by or through.

The presence of trees was also well-appreciated. Furthermore, participants found it pleasant to have a look at the front gardens on their route. However, it should be noted that participants enjoyed these naturalistic elements more during spring, summer and autumn compared to winter.

Next to the presence of vegetation, the presence of animals (e.g. birds, sheep, squirrels…) was considered pleasant as well.

##### Noise and smell

Some participants mentioned that they like to walk in quiet and calm streets and dislike to walk in streets with noise and exhausts of cars.

##### Openness

**Figure 6:**
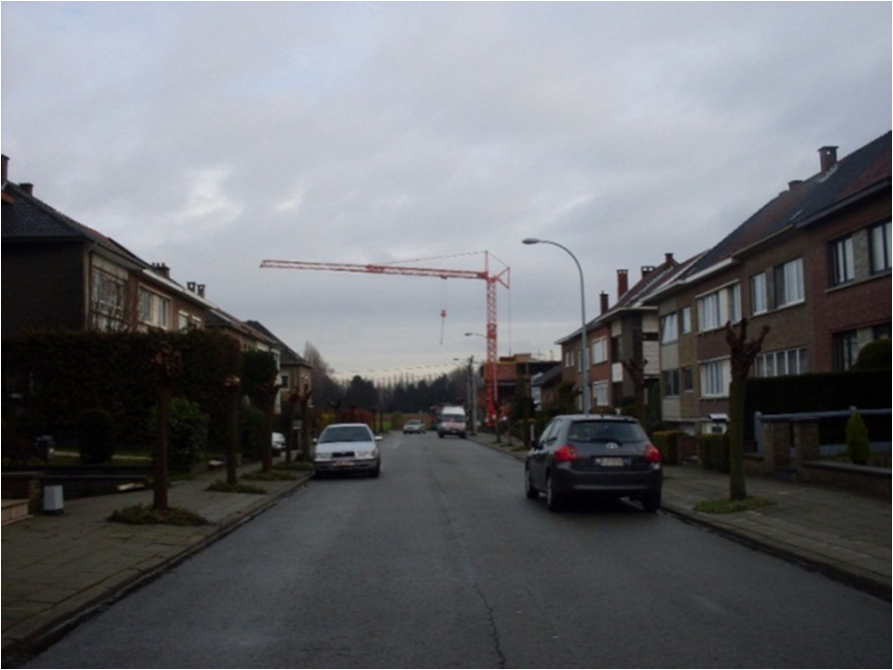
A street with an open view that is getting filled with buildings.

Few participants reported preferring open rather than closed views during their walks. Openness was associated with linearity and width of the street and absence of (high-rise) buildings. For example, a 77 year-old-man complained:

"*You could see till the end of the street, but steadily the view gets filled with buildings. Until two months ago, you had a wide and green view…* (see Figure 6)*’*"

##### Decay

Some participants liked walking in clean places and disliked the presence of garbage in streets and parks. They complained about people throwing litter in the streets/parks despite the presence of garbage cans. Furthermore, well-maintained houses and gardens made a street more attractive for walking, whereas abandoned and worn-out buildings were mentioned as making streets unattractive.

#### Weather

Some participants talked about weather conditions influencing their walking for transportation. Several of the previously described environmental factors are influenced by weather and seasonal conditions. Greenery was reported to be more beautiful during spring and summer because of the presence of leaves and flowers on trees and bushes. Wintertime was associated with perceived lack of safety from crime because of early darkness and with fear of falling because of icy and snowy conditions. Rainy weather was disliked by the participants because it produces puddles on sidewalks and mud on shoulders and field tracks. However, few participants (8.8%) also mentioned that they would walk instead of cycle for transportation during rainy weather because it is possible to carry an umbrella while walking.

#### Relative importance of environmental factors

The presence of certain environmental factors appeared to be more important than the presence of other factors. The presence of a “negative” factor might even outweigh the effect of a “positive” factor. Such an interaction between different factors is illustrated by a 73-year-old woman’s statement:

"“*There’s a lot of traffic over here. Especially during the morning, then here’s a long traffic congestion. I prefer a bad and uneven path without traffic compared to this even sidewalk with all this traffic. I always avoid the traffic as much as possible, even when the alternative paths are bad, muddy or whatever*.”"

#### Differences between men and women

Overall the same environmental factors were discussed by male and female participants. However, there were some marked differences in frequencies of discussing certain environmental characteristics between male and females (see Table 3). For example, issues related to familiarity and safety from crime were more intensely and frequently described by women.

## Discussion

The current study used walk-along interviews to investigate the perceived environmental factors influencing walking for transportation among Flemish older adults. This novel method resulted in detailed and context-specific insights in the influence of previously studied and new environmental factors.

Our finding that good access to facilities (i.e. shops and services) encourages walking for transportation supports results from previous qualitative [20,22,23,25] and quantitative studies [35,40,41]. Findings from the current study suggest that convenience (short distances) might not be the only explanation why the presence of facilities promotes walking for transportation. The presence of other people in shops and shopping streets might as well evoke feelings of safety from crime and provide opportunities for social contacts. Furthermore, our participants enjoyed looking at the show windows of shops. When designing new neighborhoods or senior housing, planners should foresee facilities within walkable distances from the residences. Integrating shops and services into existing neighborhoods might be more difficult to establish. However, the disappearance of local shops and services should be avoided as this might negatively affect walking for transportation among older adults.

Another category of environmental factors which are more amenable to change are walking facilities. Sidewalks were present in most of the streets walked along. However, almost all participants complained about the sidewalks’ quality. In line with previous qualitative studies, elements encompassing an increased risk of falling (e.g. uneven sidewalks, cracked tiles, snow…) were feared by our participants [20,21,24,25]. Additionally, in the current study the width of the sidewalks and separation of the sidewalks from streets and cycling paths appeared to be important components of sidewalk quality. Next to the presence of well-maintained sidewalks the provision of safe crossings (e.g. zebra crossings, traffic lights…) emerged as an important factor. This latter is also in support of previous qualitative studies [20,21,23,25].

Traffic safety, encompassing the subcategories “busy traffic” and “behavior of other road users”, emerged as a major issue during the walk-along interviews. In agreement with previous qualitative studies [20,23,25] participants preferred walking along streets with little traffic. This is in contrast to the positive relationship between objectively measured traffic volume and utility of a street section for transportation walking among older adults reported by Borst et al. [42]. A possible explanation for this contradiction might be that important walking destinations (e.g. shops) are often located in busy streets. Consequently, although older adults prefer to walk in streets with little traffic, they are often obliged to walk in more busy streets in order to reach their destinations. Next to busy traffic, speeding traffic emerged as another source of traffic insecurity and dislike. This is consistent with previous qualitative research [20,25]. Similar to findings by Grant et al. [23], our participants did not only fear careless car drivers but also careless cyclists. This explains the importance of sidewalks being clearly separated from cars and cyclists as described above. No quantitative study has examined the relationship between fear from collisions with cyclists and walking for transportation yet.

The presence of other people did not only influence feelings of safety from crime but was also liked because it provides opportunities for social contact. Gallagher et al. [24] concluded that the presence of familiar and friendly people in the neighborhood does influence walking, whether or not these people are engaging in PA themselves. This is supported by a quantitative study in the US which reported neighborhood social cohesion to be positively related to neighborhood walking [43]. In the current study, the presence of other people was stated to facilitate walking for transportation, while the presence of a large crowd or youngsters displaying antisocial behavior possibly has the opposite effect. In the context of ecological models, research has primarily focused on the relationship between the physical environment and PA and less on the social environment. Based upon the above described findings, future studies investigating the relationship between the social environment and older adults’ walking for transportation should be encouraged.

Concerning aesthetics, in accordance with previous qualitative studies, noiseless, clean and well-maintained streets with attractive sights (e.g. historic buildings) or natural elements were perceived as attractive to walk through by our participants [21,23,24].

Weather conditions were discussed across the categories walking facilities, safety from crime and aesthetics. The quality of walking facilities was reduced by rain causing puddles and mud and by snow and ice causing danger to slip and fall. The increase in fear of falling during snowy and icy conditions was also thoroughly described in a Canadian study using a photovoice methodology [25]. However, during data collection the winter was harsh in Belgium and the influence of snow and ice might have been less apparent when data were collected during another winter. Early darkness and diminished greenery are two other reasons why the winter was the least liked season for walking. These factors might explain the lower PA rates observed during fall and winter [44].

Although participants discussed several environmental factors separately, our findings also point to the importance of combinations of factors. Studying adults, Sallis et al. [45] reported that the presence of at least four favorable environmental factors is required to find a significant relationship with PA. In addition, our results suggest that the anticipated positive influence of certain factors (e.g. the presence of trees and high-quality sidewalks) might be outweighed in the presence of a negative factor (e.g. busy traffic). Furthermore, some environmental factors might influence walking for transportation stronger than others. Possibly, this is partially explained by the influence of some environmental factors on walking for transportation through several ways. For example, busy traffic has an effect on traffic safety but also on aesthetics through increasing cars’ exhausts. The same environmental factor might also simultaneously exert a positive and negative influence on the attractiveness for walking for transportation. For example, the presence of greenery might increase the aesthetic appeal of a street but possibly also provides hiding places for potential offenders which might increase feelings of insecurity. Interactions between and possible unanticipated effects of environmental factors have not yet been studied but warrant attention in future research.

Findings from the current and previous qualitative studies are clearly not confirmed by quantitative studies. Whereas qualitative studies consistently point to the importance of high-quality walking facilities, safety from traffic and crime and aesthetics, findings from quantitative studies are inconsistent [14]. First, this might be explained by difficulties in measuring environmental perceptions and walking behaviors (e.g. how to define an older adults’ neighborhood in which perceptions should be assessed?). Additionally, it might be difficult to capture perceptions of environmental factors when the participants are not simultaneously exposed to these factors. This was overcome in the current study by conducting walk-along interviews and might explain the obtained richness and detail in data. Second, as described above, the absence of statistical relationships in previous quantitative studies might result from investigating environmental factors individually rather than in combination or in interaction with one another.

Future research could benefit from walk-along interviews for several reasons. First, they provide rich, detailed and context-specific information on how and why previously studied environmental factors influenced walking for transportation among older adults. Second, they revealed “new” (not yet studied) environmental factors of relevance to older adults’ PA behaviors (i.e. openness, hiding places and familiarity). Third, the participants appreciated the use of walk-along interviews, which, compared to traditional interviews, create a more egalitarian relationship between researcher and participant [26,27]. Fourth, the vivid stories linked to certain neighborhoods or situations can help convince policy makers to make the environmental changes to promote PA.

An additional strength of the current study is the relatively large sample size which led to a saturation of information. Furthermore, the study captured a large sample of the environment; three different (semi-)urban regions and two different walking routes per participant. Hence, participants were exposed to a wide variety of environmental factors.

Regarding the limitations, as the study was conducted in fall and winter, the degree to which similar results would emerge in spring or summer is unclear. Because our sample consisted of (semi-)urban dwelling, highly educated, active and functionally fit older adults, one should also be cautious in extrapolating the results to rural dwelling, less educated, less active or functionally impaired older adults. For example, busy traffic might be less an issue in rural areas and quality of sidewalks might be even more important in functionally impaired older adults with great fear of falling. Furthermore, since the (semi-)urban environmental context differs between cities, countries or continents findings of the current study are not necessarily generalizable to other regions. More research is definitely needed to test how well our results apply to other seasons and subgroups in different parts of the world.

In conclusion, our findings indicate that in order to promote walking for transportation among older adults, a neighborhood should provide good access to shops and services, well-maintained walking facilities, aesthetically appealing places, streets with little traffic and places for social interaction. In addition, the neighborhood environment should evoke feelings of familiarity and safety from crime. The on-site exposure of participants enabled us to collect detailed information about specific environmental factors contributing to the above described conditions of an “ideal neighborhood” to walk for transportation. Future quantitative studies should investigate if (changes in) these environmental factors are related to (changes in) older adults’ walking for transportation.

## Competing interests

The authors declare that they have no competing interests.

## Authors’ contributions

JVC, VVH, PC, LG, JN, JS, IDB and BD developed the study design. JVC, VVH, DS and RD conducted the data collection. JVC, DS, RD and BD performed the data analysis. JVC drafted the manuscript and all other authors critically reviewed and revised versions of the manuscript. All authors read and approved the final manuscript.
